# β-Secretase1 biological markers for Alzheimer’s disease: state-of-art of validation and qualification

**DOI:** 10.1186/s13195-020-00686-3

**Published:** 2020-10-16

**Authors:** Harald Hampel, Simone Lista, Eugeen Vanmechelen, Henrik Zetterberg, Filippo Sean Giorgi, Alessandro Galgani, Kaj Blennow, Filippo Caraci, Brati Das, Riqiang Yan, Andrea Vergallo, for the Alzheimer’s Precision Medicine Initiative (APMI), Mohammad Afshar, Lisi Flores Aguilar, Leyla Akman-Anderson, Joaquín Arenas, Jesús Ávila, Claudio Babiloni, Filippo Baldacci, Richard Batrla, Norbert Benda, Keith L. Black, Arun L. W. Bokde, Ubaldo Bonuccelli, Karl Broich, Francesco Cacciola, Filippo Caraci, Giuseppe Caruso, Juan Castrillo, Enrica Cavedo, Roberto Ceravolo, Patrizia A. Chiesa, Massimo Corbo, Jean-Christophe Corvol, Augusto Claudio Cuello, Jeffrey L. Cummings, Herman Depypere, Bruno Dubois, Andrea Duggento, Enzo Emanuele, Valentina Escott-Price, Howard Federoff, Maria Teresa Ferretti, Massimo Fiandaca, Richard A. Frank, Francesco Garaci, Hugo Geerts, Ezio Giacobini, Filippo S. Giorgi, Edward J. Goetzl, Manuela Graziani, Marion Haberkamp, Marie-Odile Habert, Britta Hänisch, Harald Hampel, Karl Herholz, Felix Hernandez, Bruno P. Imbimbo, Dimitrios Kapogiannis, Eric Karran, Steven J. Kiddle, Seung H. Kim, Yosef Koronyo, Maya Koronyo-Hamaoui, Todd Langevin, Stéphane Lehéricy, Pablo Lemercier, Simone Lista, Francisco Llavero, Jean Lorenceau, Alejandro Lucía, Dalila Mango, Mark Mapstone, Christian Neri, Robert Nisticò, Sid E. O’Bryant, Giovanni Palermo, George Perry, Craig Ritchie, Simone Rossi, Amira Saidi, Emiliano Santarnecchi, Lon S. Schneider, Olaf Sporns, Nicola Toschi, Pedro L. Valenzuela, Bruno Vellas, Steven R. Verdooner, Andrea Vergallo, Nicolas Villain, Kelly Virecoulon Giudici, Mark Watling, Lindsay A. Welikovitch, Janet Woodcock, Erfan Younesi, José L. Zugaza

**Affiliations:** 1 Sorbonne University, GRC no 21, Alzheimer Precision Medicine (APM), AP-HP, Pitié-Salpêtrière Hospital, Paris, France; 2 Brain & Spine Institute (ICM), INSERM U 1127, CNRS UMR 7225, Boulevard de l’hôpital, F-75013 Paris, France; 3 Institute of Memory and Alzheimer’s Disease (IM2A), Department of Neurology, Pitié-Salpêtrière Hospital, AP-HP, Boulevard de l’hôpital, F-75013 Paris, France; 4 ADx NeuroSciences NV, Technologiepark 4, 9052 Ghent, Belgium; 5 Department of Psychiatry and Neurochemistry, Institute of Neuroscience & Physiology, the Sahlgrenska Academy at the University of Gothenburg, Mölndal, Sweden; 6 Clinical Neurochemistry Laboratory, Sahlgrenska University Hospital, Mölndal, Sweden; 7 Department of Neurodegenerative Disease, UCL Institute of Neurology, Queen Square, London, UK; 8 UK Dementia Research Institute at UCL, London, UK; 9 Human Anatomy, Department of Translational Research and New Technologies in Medicine and Surgery, University of Pisa, Pisa, Italy; 10 Department of Clinical and Experimental Medicine, University of Pisa, Pisa, Italy; 11 Department of Drug Sciences, University of Catania, Catania, Italy; 12 Oasi Research Institute-IRCCS, Troina, Italy; 13 Department of Neuroscience, University of Connecticut Health, Farmington, CT USA

**Keywords:** Alzheimer’s disease, Amyloid-β pathway, Axonal damage, BACE1, Clinical trials, Context of use, Fluid biomarkers, Neurodegeneration

## Abstract

β-Secretase1 (BACE1) protein concentrations and rates of enzyme activity, analyzed in human bodily fluids, are promising candidate biological markers for guidance in clinical trials investigating BACE1 inhibitors to halt or delay the dysregulation of the amyloid-β pathway in Alzheimer’s disease (AD). A robust body of evidence demonstrates an association between cerebrospinal fluid/blood BACE1 biomarkers and core pathophysiological mechanisms of AD, such as brain protein misfolding and aggregration, neurodegeneration, and synaptic dysfunction.

In pharmacological trials, BACE1 candidate biomarkers may be applied to a wide set of contexts of use (CoU), including proof of mechanism, dose-finding, response and toxicity dose estimation. For clinical CoU, BACE1 biomarkers show good performance for prognosis and disease prediction.

The roadmap toward validation and qualification of BACE1 biomarkers requires standardized pre-analytical and analytical protocols to reduce inter-site variance that may have contributed to inconsistent results.

BACE1 biomarker-drug co-development programs, including biomarker-guided outcomes and endpoints, may support the identification of sub-populations with a higher probability to benefit from BACE1 inhibitors with a reduced risk of adverse effects, in line with the evolving precision medicine paradigm.

## Introduction

**Fig. 1:**
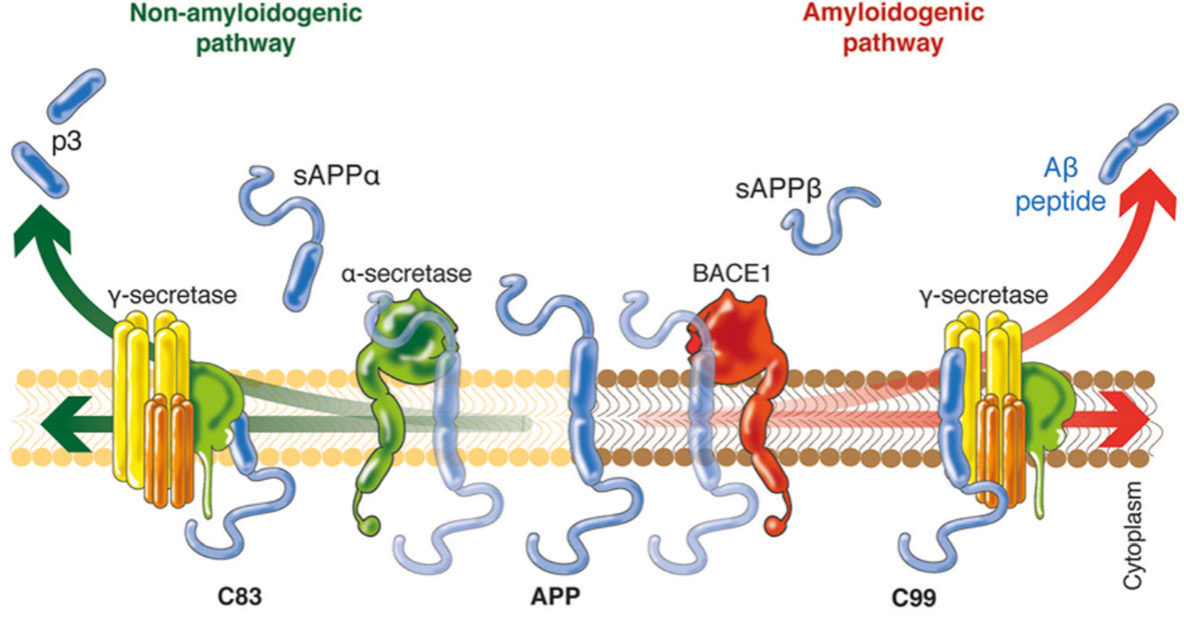
Schematic representation of amyloidogenic and non-amyloidogenic pathways. Footnote: Three main proteases—α-, β-, and γ-secretases—are involved in APP processing through the amyloidogenic pathway (sequential cleavage by β- and γ-secretases), promoting amyloid-β (Aβ) production, and the non-amyloidogenic pathway in which Aβ is cleaved in the middle, either directly by α-secretase (generating soluble APPα) or by the sequential cleavage by β-secretase and α-secretase (generating shorter Aβ species such as Aβ1–15 and Aβ1–16). The two pathways lead to the production of different by-products with different intrinsic functional properties, putative physiological roles, and pathophysiological potential. In particular, BACE1 serves as the β-secretase enzyme by cleaving the transmembrane APP to release the β-stubs. BACE1 cleavage of APP represents the rate-limiting step for Aβ production. Cleavage of APP by BACE1 liberates the soluble N-terminus of APP, while the C-terminal fragment (CTF-β or C99) remains bound to the membrane. To produce Aβ, the fragment CTF-β is cleaved by γ-secretase, an aspartyl-type protease membrane protein complex, which finally releases Aβ into the extracellular space and the APP intracellular domain into the cytoplasm. The γ-secretase consists of different components. The catalytic components of the membrane-embedded tetrameric γ-secretase complex are represented by presenilins 1 and 2, intramembrane-cleaving proteases (I-CLIPs), responsible for generating the Aβ carboxyl terminus from APP. In a parallel competing non-amyloidogenic pathway, APP is cleaved either by α-secretase or η-secretase to release two additional variants of the APP ectodomain, namely sAPP-α and sAPP-η. In vitro studies have shown that ADAM-10, a disintegrin and metalloprotease belonging to the family proteases, is the major α-secretase responsible for the ectodomain shedding of APP in the mouse brain and likely to be active in humans. APP is a type I transmembrane protein, highly expressed in neurons and abundant at the synapse. Although a full understanding of its function remains elusive, studies have suggested a role in the remodeling of dendritic spines, neurotransmission, synaptic plasticity, and maintenance of excitation-inhibition (E/I) balance. Soluble sAPPα and sAPPβ are hypothesized to modulate basal synaptic transmission and short-term synaptic facilitation likely through GABAB receptor subunit 1a-mediated synaptic effect. Note: Adapted from [4]. Reproduced with permission

β-Site amyloid precursor protein (APP) cleaving enzyme 1 (BACE1) is a type I transmembrane aspartyl protease widely expressed in the brain, particularly in neurons, oligodendrocytes, and astrocytes [1–3]. BACE1 is expressed at the plasma endothelial membrane and in the endosomal compartments and has been detected in healthy synaptic terminals. BACE1 functions as the β-secretase enzyme by cleaving the transmembrane APP to release the β-stubs and represents the rate-limiting catalytic step for Aβ production (see Fig. 1) [1–3].

High BACE1 concentrations (probably reflecting gene expression levels) and enzymatic activity were found in human AD brain extracts, consistent with experimental evidence that neurons express higher levels of Aβ in AD compared to “cognitively healthy aging.” In addition, a relatively large accumulation of BACE1 was found in neuritic dystrophies in close proximity of Aβ plaques both in AD amyloidogenic transgenic mouse models and in AD brains, and this presence may promote cyclic Aβ production [3, 5, 6].

Although BACE1 mutations have not yet been linked to AD risk, genetic variants surrounding the β-secretase site in the APP (including the Swedish mutation *KM/NL*, the Italian variant *A673V*, and the *A673T*) are associated with either higher or lower affinity for BACE1 to initiate APP cleavage, thus exerting a protective or risk effect, respectively [3].

The reported translational results provide robust proof of principle for the pathophysiological and pharmacological model, indicating that reducing the β-cleavage of APP may be a resilience mechanism for AD [3].

With the advent of oral and blood-brain barrier (BBB)-permeable inhibitors, BACE1 has become a central target in several drug AD R&D pipelines. Despite intense pharmacological efforts, all clinical trials so far have been discontinued for futility or signs of cognitive worsening or some systemic toxic effects, thus raising relevant safety and efficacy concerns [7, 8].

One of the most significant issues after a first reflection on discontinued clinical trials is that they did not introduce any direct BACE1 biomarkers for any relevant context of use (CoU), such as proof of mechanism, dose-finding, and efficacy/safety measures. Implementation of existing BACE1 biomarkers would support the mapping of drug response, optimization of go/no-go decision-making, and mitigation of side effects due to non-specific or too high BACE1 inhibition [3].

## Search strategy and selection criteria

The narrative inherent to this review article is based on the authors’ knowledge and experience in the field. As such, no systematic literature search was performed.

## BACE1 biomarkers in the cerebrospinal fluid (CSF)

BACE1 protein concentrations, probably reflecting levels of gene expression, and rates of enzymatic activity, have been measured in human CSF samples to investigate their diagnostic/predictive values as well as their association with critical pathophysiological alterations of AD, including the amyloid-β pathway, tau pathophysiology, neurodegeneration, and synaptic dysfunction [3].

### CSF BACE1 biomarker: diagnostic and predictive performance

The first study analyzed BACE1 CSF concentrations and activity in a pooled cohort of cogntively healthy control (HC) individuals, clinically diagnosed patients with AD dementia (ADD), and individuals with mild cognitive impairment (MCI) [9].

The authors reported that individuals in which BACE1 both enzymatic activity and protein levels are in the higher ranges showed an increased relative risk of association with the MCI group when compared to HC or ADD individuals. The finding of elevated BACE1 biomarkers in individuals with MCI compared to ADD was discussed in relation to extensive dendritic remodeling and neuronal loss characterizing dementia stages [9].

Zetterberg and colleagues investigated BACE1 activity in a comparable pooled cohort, reporting a statistically significant difference between patients with ADD and HC individuals, but not between ADD patients and a diagnostic group of HC combined with MCI individuals [10]. When further differentiating the group of MCI individuals into ADD converters and non-converters, they found higher mean BACE1 activity in the former subgroup than in the latter one [10]. Such results were corroborated in subsequent studies [11–13].

In a separate study, Perneczky and colleagues assessed BACE1 activity in a population of 342 individuals, including HC, stable MCI, converted MCI individuals, and ADD patients. Contrary to these findings, no significant differences in BACE1 activity were found between the investigated groups. The authors argued that such unexpected results could be partly due to the different used assays, which may have impacted BACE1 activity due to higher intra-assay variability [14].

Likewise, Savage and colleagues found no significant difference regarding BACE1 activity between individuals with HC, MCI, and ADD. In line with the argumentation of Perneckzy and colleagues, they hypothesized that the wide inter-subject variability of BACE1 activity along with technical differences of laboratory assays could have limited the analytical standardization and clinical validation of the CSF BACE1 diagnostic candidate biomarker [15].

### CSF BACE1 and amyloid-β biomarkers

Studies investigating CSF BACE1 biomarkers and indicators of brain accumulation of Aβ, including CSF 42-amino acid forms of amyloid-β protein (Aβ42) and Aβ positron emission tomography (Aβ-AΒ-PET), do not show consistent results, as some studies did not find any significant association [10, 14, 16, 17], while other studies showed significant correlations [18–20].

A significant association was found in studies that stratified the whole study population according to the clinical diagnosis, reporting positive correlations only in HC individuals and patients with ADDs [18–20], but not in individuals with MCI [18]. There is no clear biological explanation for the variation of results across different studies but rather a potential methodological issues related to employing different study designs, populations, and assays. The diverging results (i.e., Aβ42 is a product of the BACE1 pathway and thus an association was expected) have generated some discussion.

Some authors raised the question of whether Aβ42 monomers truly provide comprehensive information on the whole Aβ pathway that encompasses small aggregation species, oligomers, protofibrils, fibrils, and eventually senile plaques [3, 10]. Effective clearance of Aβ aggregates will impact the concentrations of Aβ42 in CSF as well. Therefore, the whole unfolding of the amyloid-β pathway can account for a non-linear association between BACE1 and Aβ42 monomers (see below). Despite the lack of a robust correlation of CSF Aβ42 with BACE1 concentrations, a multimodal study showed that CSF BACE1 activity is correlated with a global uptake of the Pittsburgh Compound B PET (PiB-PET) tracer, a radiotracer that binds to the fibrillary component of amyloid plaques [21].

BACE1 CSF parameters correlate, at least in part, with other Aβ markers. In particular, a strong positive correlation between BACE1 and levels of Aβ40, sAPP-α, and sAPP-β has been reported. Interestingly, although sAPP-α is a by-product of the alternative pathways of α-secretase, it negatively correlates with BACE1 [10]. This finding may be explained through collinearity between sAPP-α and sAPP-β that are highly associated with one another or by the fact that both by-product may reflect the rate of APP processing [10].

### CSF BACE1 and biomarkers of tau-related pathophysiology

Concerning the association between BACE1 and tau-related pathophysiology, multiple groups—using different methodological approaches and study designs—found a positive correlation between CSF BACE1 biomarkers and CSF tau phosphorylated at threonin181 (t-tau and p-tau, respectively) [22].

Experimental evidence and translational studies can help explain the association between p-tau and the amyloid-β pathway, including the putative upstream role of BACE1. Indeed, injection of Aβ fibrils into the brains of P301L mutant tau transgenic mice triggers a fivefold increase in NFTs in cell bodies within the amygdala where neurons project to the injection sites [23]. In another study, crossing transgenic mice showing the spread of tau from the entorhinal cortex to other brain regions with APP/PS1 mice showed that cortical amyloid deposition caused a dramatic increase in tau spreading to distal brain regions. Hence, several findings point toward an upstream role of Aβ, and on an inferring speculative basis BACE1, on tau phosphorylation and neurofibrillary tangle generation by facilitating and promoting the conversion of tau from a normal to a toxic state, which may enhance Aβ toxicity via a feedback loop [24, 25]. Such experimental evidence supports the data-driven (biomarker-based) hypothetical model of AD clinical-biological continuum whereby brain accumulation of Aβ may either facilitate being permissive to spreading of tau pathology that is tightly associated with the clinical evolution of the disease [22].

### CSF BACE1 biomarkers and neurodegeneration

CSF BACE1 biomarkers have been investigated in relation to hippocampal volume loss, a biomarker of regional neurodegeneration occurring during early stages of AD.

The only published structural MRI study reported that an increase in CSF BACE1 activity is associated with bilateral decreased hippocampus volume [11]. The interpretation of this finding is unclear; however, it may suggest a BACE1-mediated neurotoxicity. The observed BACE1 activity in CSF inversely correlating with hippocampal volume supports the hypothesis that elevated BACE1 may induce downstream amyloidogenic effects by triggering stepwise neurodegeneration leading to hippocampal atrophy.

Evidence indicating an association between BACE1 and neurodegeneration can be derived from studies reporting positive correlations between CSF BACE1 biomarkers and CSF total tau protein [9–12, 14–20, 26], a surrogate marker of axonal damage and neuronal loss. It is conceivable that BACE1 is released into CSF by degenerating neurons and that the concentrations may correlate with the severity of neurodegeneration and the progression of synaptopathy and neuronal loss. Dysregulation of synaptic BACE1 functions may account in part for a non-amyloidogenic impact on synaptic homeostasis.

Beyond any preliminary data-driven and knowledge-based consideration, it must be outlined that major parts of evidence regarding the association between BACE1 and tau biomarkers in CSF have been studied cross-sectional. Longitudinal observational studies are needed to investigate the spatial-temporal relationship between BACE1 biomarker expression, gene expression levels and activity, and neurodegeneration.

By contrast, the results of the phase 3 trial of verubecestat (12 or 40 mg/day) conducted in mild-to-moderate AD patients (EPOCH, ClinicalTrials.gov NCT01739348) showed significant BACE1 inhibition correlating with decreased hippocampal volumes [3]. Although a univocal interpretation of this trial result is challenging, it has been hypothesized that there is an over inhibition of BACE1 regulation of synaptic substrates and or physiological functions of Aβ species, essential also for hippocampal homeostasis [3] (see Fig. 1 for more details about the amyloidogenic pathway). Follow-up analysis is needed in BACE1 inhibitor trials to ascertain whether this hippocampal effect is related to the cognitive worsening reported in some studies and whether it may reversible.

### CSF BACE1 and synaptic biomarkers

Two studies investigated the association between CSF BACE1 and biomarkers of synaptic dysfunction. De Vos and colleagues analyzed CSF BACE1 and neurogranin (NGR)—a dendritic protein proposed as a biomarker of hippocampal synaptic impairment [27]—in a pooled cohort of HC individuals HC and positive Aβ biomarkers individuals diagnosed with MCI or ADD [18]. Despite no significant inter-group differences, they found that the NGR/BACE1 ratio differentiates both the MCI and ADD diagnostic groups from the HC individuals group with good accuracy [18]. The NGR/BACE1 ratio also showed potential prognostic value since individuals with higher concentrations had a more severe cognitive decline at follow-up [18].

In agreement with De Vos and colleagues, a recent report showed that NGR/BACE1 ratio levels are (i) elevated in both individuals with subjective cognitive decline (SCD) and MCI compared to HC individuals, (ii) associated with smaller hippocampal and amygdala volumes, and (iii) correlate with worse baseline and longitudinal cognitive performance [28].

A recent study investigated a broad set of candidate biomarkers tracking distinct pathophysiological processes in patients with AD and reported a positive cross-sectional correlation between BACE1 CSF concentrations and NGR [20]. Interestingly, another group reported that the CSF NGR/BACE1 ratio, along with core AD biomarkers, displays good accuracy to distinguish between depression with cognitive impairment and AD dementia [29].

Given the established neurobiological overlap among depression, MCI, and AD, the NGR/BACE1 ratio may represent a suitable clinical tool for the AD diagnostic workup [30]. Further prospective longitudinal studies are needed to understand the role of the NGR/BACE1 ratio as a biomarker to improve the classification between non-neurodegenerative forms of MCI, including but not exclusively depression, and early AD.

The understanding of the relationship between BACE1 and synaptic homeostasis remains an unmet objective. There is evolving experimental evidence, i.e., data from conditional deletion of BACE1 in mouse models, that points at BACE1 as a molecular orchestrator of hippocampal synaptic remodeling at the dendritic and axonal level [3, 7]. Arguably, BACE1 overactivation may excessively accelerate synaptic turnover until it triggers downstream detrimental pathways resulting in synaptic damage. The generated hypothesis is further supported by studies investigating the association between BACE1 and other candidate biomarkers of neurodegeneration and synaptic loss.

### CSF BACE1 biomarkers and the *APOE ε4* allele

Ewers and colleagues reported an association of the apolipoprotein E (*APOE) ε4* allele with increased BACE1 activity in both patients with ADD and subjects with MCI compared to HC individuals [31]. This finding is in agreement with experimental models of AD, indicating increased activity of BACE1 in individuals carrying the *APOE* ε4 allele [32]. It is unclear whether this correlation is induced by *APOE ε4* allele in promoting Aβ deposition, which subsequently can induce increase of BACE1 activity. Consistently, in-human neuropathological studies show higher concentrations of BACE1 in HC individuals or ADD patients, carrying the *APOE ε4 allele* [33]*.* Two independent studies did not show any association between the *APOE* genotype and BACE1 concentrations or rates of activity. Of note, the two studies differed significantly with each other and compared to other investigations, in terms of experimental design, including the assay utilized [15, 16]; see the section “Potential explanation of controversial results in CSF (and blood-based) BACE1 studies” for a more in-depth argumentation.

CSF-based studies provide evidence that BACE1 biomarkers, both protein concentrations and enzymimatic activity, support further analytical and clinical investigations in patients with AD to investigate their potential as candidate biomarkers suitable for clinical practice (i.e., early diagnosis, prediction, and progression) and pharmacological trials targeting BACE1 (i.e., target engagement and efficacy response, among others). Further studies with longer follow-up, standardized pre-analytical procedures, and analytical protocols are required to address the open questions based on conflicting study data.

## BACE1 blood-based biomarkers

### BACE1 biomarkers in plasma

While BACE1 is primarily expressed in the CNS, the protease is expressed in platelets, leukocytes, and is circulating as a soluble protein in the plasma.

For different CoU, blood-based biomarkers provide unique opportunities and decision-making tools in clinical trial programs. Blood-based biomarkers have numerous advantages, i.e., they are widely accessible and minimally invasive and are more time- and cost-effective for healthcare systems compared with CSF. In particular, they are appropriate tools to inform biomarker-guided medicine applied to individuals with preclinical AD [27].

Both BACE1 activity and protein concentrations in blood (mostly plasma but in some cases serum) are significantly increased in MCI individuals or patients with ADD compared to HC individuals, with a trend across different disease stages reflecting the direction of expression of the reported CSF biomarkers [34–36].

Shen and colleagues reported a study in which both blood BACE activity and protein concentration were measured and explored in parallel with correlations of CSF AD core biomarkers [36]. The population included individuals with ADD, HC, MCI converters to AD, and MCI stable at follow-up. They showed that BACE1 activity was elevated in both individuals with ADD and MCI converters when compared to stable MCI or HC individuals; at the same time, BACE1 protein concentrations were significantly increased in individuals with ADD compared to HC or stable MCI, while BACE1 concentrations in converter MCI individuals were significantly elevated compared to subjects with stable MCI, but not compared to the HC group [36]. BACE1 activity was positively correlated with CSF t-tau protein and negatively correlated with CSF Aβ42, further supporting a link between the plasmatic biomarker and brain AD pathology [36].

A recent plasma-based study tested associations between plasma BACE1 concentrations and the degree of cerebral accumulation of Aβ in a cohort of HC with subjective memory complaints (SMC), a condition associated with  increased risk for AD. For this objective, brain accumulation of Aβ was investigated using Aβ positron emission tomography (PET) imaging (Aβ-PET) [37] showing, for the first time, that plasma BACE1 concentrations impact the level of brain Aβ in individuals with SMC [37].

The same study further investigated the question of whether other relevant biological factors, such as sex, besides the APOE ε4 allele and age, may affect plasma BACE1 concentrations [37]. They found highly significantly increased baseline and longitudinal mean concentrations of BACE1 in women compared to men, irrespective of age and time [37]. These results indicate a potential sexual dimorphism in plasma BACE1 concentrations, in agreement with experimental evidence about the role of estradiol in the control of BACE1 expression [38].

### BACE1 assessed in platelets

Studies conducted in platelets show an increase of BACE1 protein concentrations and higher activity rates in individuals with ADD and or MCI compared to HC [39–41]. One study did not find differences between individuals with HC and MCI, while Decourt and colleagues found decreased BACE1 protein concentrations in individuals with ADD when compared to HC [42].

Another platelet-based study reported that patients with ADD treated with a stable 6-month dose of donepezil, but not cognitively healthy controls, showed downregulation of BACE1 gene expression in platelets [43]. Given the consistency between the study results and AD pathophysiology, including BACE1 overexpression, the authors argued that BACE1 platelet levels should be investigated to ascertain whether they might represent an additional exploratory outcome measure to employ in BACE1 inhibitor trials.

### BACE1 transcriptomic studies in blood

Besides assessing enzymatic biomarkers, molecular biology investigations of BACE1 have used blood samples. In particular, three studies explored the variability of BACE1 gene expression in AD. In 2019, Wongchitrat and colleagues measured the rate of BACE1 mRNA expression in peripheral leukocytes and showed significantly higher mRNA levels in ADD patients compared to HC individuals [44]. In the same year, Vakilian and colleagues obtained similar results, and in their extension study, they assessed BACE1 concentration in using the same blood samples as in the genetic research. Although they found increased concentrations of BACE1 in ADD patients, they did not observe any correlations between BACE1 and mRNA expression [45].

A different approach was chosen by Fotuhi and colleagues, which evaluated circulating long noncoding RNA (lncRNA) related to the BACE1 gene (BACE1-AS) in plasma and plasma-derived exosomes. The lncRNA BACE1-AS is believed to improve BACE synthesis, via mRNA stabilization [46].

They did not observe any difference in exosome lncRNAs; however, they found that lcRNA BACE1-AS plasmatic concentration was significantly lower in mild ADD compared to HC individuals, differentiating HC individuals from mild ADD with good sensitivity and specificity [46].

## Potential explanation of controversial results in CSF (and blood-based) BACE1 studies

 Inconsistent results complicate clinical validation and qualification of BACE1-related biomarkers and their potential integration into the evolving AD biomarker matrix. Existing discrepancies need to be carefully scrutinized to see whether methodological issues, rather than biological implications, may determine differences.

First, as indicated by human neuropathological and experimental models of aging and AD, BACE1 gene expression and rates of activity may vary throughout AD progression. In this context, Rosen and colleagues found that BACE1 activity was significantly increased in AD patients with mild dementia compared to patients at more severe stages [12]. A recent study showed that BACE1 biomarker candidates are significantly increased in individuals with MCI, but not with ADD, when compared with the HC group [13]. Therefore, it is likely that the disease stage of the individual patient may influence BACE1 concentration and activity, and thus, clinical heterogeneity of included individuals may have neutralized inter-group differences.

Second, since BACE1 had been proposed as a potential AD-specific biomarker, a considerable part of the reported studies, in particular the older publications, were performed in clinically, but not biologically, characterized study populations. Hence, it is not possible to rule out that the enrollment of individuals with non-AD pathophysiology may have biased the data and created conflicting outcomes.

Most of the recent studies used CSF core biomarkers of AD, where BACE1 correlated indeed with Aβ and tau markers. In particular, Mulder and colleagues found that BACE1 activity was increased in individuals showing characteristic AD biological features compared to individuals with negative AD biomarkers [16], while Alexopoulos and colleagues showed significantly decreased CSF BACE1 activity in individuals with MCI without AD pathophysiology compared to patients with MCI due to AD [13].

Third, some studies did not report any association between CSF BACE1 biomarkers and CSF Aβ42 raising questions about the potential of BACE1 biomarkers in AD pharmacological trials. However, given the tendency of Aβ42 monomers of aggregating into oligomers and fibrils and preliminary evidence that BACE1 is associated with Aβ-PET radiotracer uptake, we suggest further studies using multimodal outcome measures such as PET tracers and emerging CSF candidates for the assessment of different Aβ species, including Aβ-oligomers and protofibrils [47–49].

Fourth, conflicting data may be partly explained by sexual dimorphism in BACE1 biology. Indeed, in a study conducted in patients suffering from bipolar disorder, a sex-based dimorphism in BACE1 gene expression levels was reported, with men displaying upregulation of BACE1 expression [50].

Furthermore, sexual dimorphism was reported in males exhibiting higher *BACE1* expression compared to females with schizophrenia and HC individuals [51].

However, female sex-biased dimorphism may exist regarding BACE1 concentrations in cognitively healthy individuals at risk for AD [37]. This finding is in line with most of the experimental evidence—mouse models of aging and AD—that indicates that intracellular effects of estrogens induce upregulation of BACE1 gene expression levels [32, 52], confirming the widely accepted notion that women bear higher vulnerability to AD [53, 54].

Apart from the necessity to elucidate in humans whether male or female sex may be a determinant of BACE1 gene expression in AD—where other genetic/biological factors may synergize with sex hormones or act independently to upregulate BACE1—evidence of sexual dimorphism in BACE1 biology may be relevant for clinical BACE1 inhibitor trial outcomes.

If a BACE1 sexual dimorphism was corroborated, sex-related outcome analyses and comparative active treatment dose-finding studies should be taken into account.

Lastly, the abovementioned CSF studies assessed different BACE1 biomarkers and some studies evaluated BACE1 enzymatic activity [10, 14, 15], while others investigated either BACE1 protein levels [18] or both biomarkers.

It should be acknowledged, however, that several in vitro and animal data point to a non-linear relationship between the levels of gene expression and rates of enzyme activity that is highly influenced by post-translational modifications [55]. Indeed, experimental studies indicate that BACE1 activity significantly increases over time while its expression levels are less likely to be altered during cognitively healthy aging as well as in the presence of AD-related cognitive decline [3].

### Methodological and technological challenges

Inconsistent results in fluid biomarker studies can derive from methodological differences.

Pre-analytical factors such as the sample collection, processing, and storage protocol, as well as analytical factors including sample handling and immunoassays used, are likely the most relevant determinants of the inter-study variability in terms of results (see Tables 1 and 2).

**Table 1:** BACE1 CSF-based biomarkers

BACE1 activity measured (method)	Antibody	Substrate	Clinical study	Result
**Sandwich ELISA**	Anti-BACE1 Ab SECB1&2 [56, 57] Anti-BACE1 Ab B280 and anti-BACE1 monoclonal Ab (R&D Systems Inc) [58]	Synthetic fluorescence substrate—containing the BACE1 cleavage site	Zhong et al. [59]	Significant elevation of BACE1 levels in MCI and AD Strong and significant correlation of BACE1 activity with BACE1 protein and Aβ peptide level
**Solution-based detection of CSF BACE activity**	Polyclonal NF neoepitope-specific Ab [60]	Biotinylated BACE1 substrate	Zetterberg et al. [10]	Significant differences in BACE1 activity between MCI, AD, and HC Positive correlation between BACE1 activity, CSF t-tau, and Aβ40 levels in the MCI and AD
**Sandwich ELISA**	Anti-BACE1 Ab SECB1&2 [56, 57] Anti-BACE1 Ab B280 and anti-BACE1 monoclonal Ab (R&D Systems Inc) [58]	Synthetic fluorescence substrate—containing the BACE1 cleavage site [59]	Ewers et al. [11]	BACE1 activity and protein levels were significantly increased in AD compared to healthy HC Increase in CSF BACE1 activity in AD Increased activity associated with increased CSF t-tau but not Aβ42 in AD
**2 step-solution-based detection of CSF BACE activity**	Anti-NF c-terminal neoepitope polyclonal Ab	Biotinylated peptide substrate (bBACE (aa1–460)) and Sapphire-II Enhancer substrate	Perneczky et al. [14] Savage et al. [15]	No significant difference in BACE1 levels between HC, MCI, and AD
**ELISA**	mAbs ADx401 (clone 5G7) and 10B8 and mAb ADx402 (clone 10B8F1) biotinylated with peroxidase [26]		De Vos et al. [18]	Significant correlation of ratio of CSF neurogranin trunc P75/BACE1 between HC, MCI, and AD
**Solution-based assay**	BACE1 Activity Assay Kit (Sigma CS1060)	BACE1 Activity Assay Kit (Sigma CS1060)	Mulder et al. [16]	No significant differences in BACE1 levels between MCI, AD, and HC
**SignalClimb technology**	Time-resolved fluorescence activity [61]	Synthetic TruePoint BACE1 substrate	Tsolakidou et al. [62]	Positive correlation between BACE1 activity and SORL1 in CSF t-tau and sAPPβ levels in AD
**Sandwich ELISA**	Anti-BACE1 monoclonalAbs (mAbs) 5G7 and 10B8 [26]		Timmers et al. [19]	CSF BACE1 correlated positively with age (weak) and with Aβ37 (strong), sAβPP-total, p-tau181 (strong)
**Commercially available assays**	Euroimmun, Luebeck, Germany		Schaeverbeke et al. [20]	Correlation of BACE1 activity with brain volumes and Aβ load in regions typically involved early in AD
**SignalClimb technology**	Time-resolved fluorescence activity [61]	Synthetic TruePoint BACE1 substrate	Grimmer et al. [21]	Strong association between BACE1 activity and in vivo Aβ pathology in brain regions close to the ventricles
**Solution-based detection of CSF BACE activity**	Polyclonal NF neoepitope-specific Ab [59]	Biotinylated BACE1 substrate	Rosén et al. [12]	BACE1 correlated slightly with sAPPα, sAPPβ, and Aβ40
**2 step ELISA**	Time-resolved fluorescence activity [61]	Synthetic TruePoint BACE1 substrate	Alexopoulos et al. [63]	No significant difference in BACE1 activity between AD and HC or MCI while BACE1 activity was significantly higher in MCI-AD compared to both HC
**Solution-based detection of CSF BACE activity**	Fluorescence-based detection in the presence of inhibitor Calbiochem	Synthetic peptide substrates containing the BACE 1 cleavage site	Ewers et al. [31]	CSF BACE1 activity significantly increased in MCI compared to AD. No significant difference between AD and HC
**BACE activity fluorometric assay kit**	K360-100, BioVision, Milpitas, CA, USA		Hou et al. [32]	BACE1 activity significantly increased in the hippocampus of ApoE4/3xTg mice especially in females
**Sandwich ELISA**	Used anti-BACE1 ectodomain MAB9311 (R&D Systems) and detection with rabbit anti-BACE1 N-terminus B0681 (Sigma–Aldrich) Abs [64]		Decourt et al. [42]	BACE1 levels 12% lower in the AD frontal cortex compared to HC 6.5% decrease in the temporal cortex
**Solution-based assay**	Used modified procaspase-3 as detection enzyme	Caspase substrate AspGluValAsp-p-nitroanilide	Verheijen et al. [65]	Assay detects BACE1 activity in extracts of human brain tissue and CSF

*Abbreviations*: *CSF* cerebral spinal fluid, *MCI* individuals with mild cognitive impairment, *AD* patients with Alzheimer’s disease dementia, *HC* cognitively healthy individuals, *ELISA* enzyme-linked immunosorbent assay, *Ab* antibody, *t-tau* total peptide tau protein, *Aβ* amyloid beta, *BACE1* beta secretase1

**Table 2:** BACE1 blood-based biomarkers

BACE1 activity (method)	Antibody	Substrate	Clinical study	Result
**ELISA**	Anti-BACE1 Ab SECB1&2 [56, 57]	Synthetic peptide substrates containing the β-cleavage site (Calbiochem, EMD, Gibbstown, NJ, USA) [59]	Shen et al. [36]	Plasma BACE1 activity significantly increased by 53.2% in MCI and by 68.9% in AD compared to HC
**ELISA**	Anti-BACE1 Ab SECB1&2 [56, 57]	Synthetic substate-C-terminally labeled with the fluorescent, Luciferase Yellow, and N-terminally labeled with the quenching, Dabsyl	Cervellati et al. [35]	Increased BACE1 activity in serum of AD
**ELISA**	Biotinylated detector mAb, diluted in a buffer adapted for the plasma matrix	Kit-based assay EQ 6541–9601-L; Euroimmun AG, Lübeck, Germany [18]	Vergallo et al. [37]	Plasma BACE1 significantly higher in women than in men in cognitively healthy individuals at clinical risk for AD
**Solution-based platelet BACE1 assay**		Fluorogenic substrate (Calbiochem, BACE1 substrate I)	Johnston et al. [39]	17% increase in platelet membrane BACE1 activity in AD compared to HC
**Solution-based platelet BACE1 assay**		Fluorescence-quenching substrate (Calbiochem, Merck, Darmstadt, Germany)	Bermejo-Bescós et al. 2013 [40]	No significant difference in BACE1 activity between MCI and HC
**Solution-based platelet BACE1 assay**		Fluorogenic substrate (Sigma A1472 or Bachem M2465)	Wongchitrat et al. [44]	Baseline platelet membrane BACE1 activity not significantly different between MCI vs HC
**Immunoassay**		Immunoassay kit (CUSABIO, USA)	Vakilian et al. [45]	Elevated plasma levels of BACE1 in AD vs HC
**RNA expression analysis**		Real-time quantitative RT-PCR	Ghafouri-Fard et al. [50]	BACE1 levels significantly high in bipolar disorder
**RNA expression analysis**		Real-time quantitative RT-PCR	Nafisi-Far et al. [51]	BACE1 levels significantly high in schizophrenia

*Abbreviations*: *CSF* cerebral spinal fluid, *MCI* individuals with mild cognitive impairment, *AD* patients with Alzheimer’s disease dementia, *HC* cognitively healthy individuals, *ELISA* enzyme-linked immunosorbent assay, *Ab* antibody, *t-tau* total peptide tau protein, *Aβ* amyloid beta, *BACE1* beta secretase1

Regarding pre-analytical factors, besides those that concern AD CSF biomarkers in general, a recent study conducted in cell lines and iPSC-derived neurons reported that 7 of the 8 BACE1 inhibitors evaluated show increased BACE1 protein concentrations [66]. A thorough pre-analytical evaluation is required to understand better the effect of BACE1 inhibitors in BACE1 biomarker assays [66].

Regarding the assessment of BACE1 biomarkers, poor specificity (i.e., other enzymatic activities may contribute to the signal) of activity-based assays may represent a plausible explanation for the observed differences [39]. In particular, peptide-based activity assays show questionable reliability for measuring BACE1 activity. To our knowledge, one of the most robust assays used is reported by Sinha and colleagues in 1999, utilizing membrane-bound substrates for measuring BACE1 activity [67]. Specifically, they purified BACE1 activity to homogeneity from human brains using a substrate analog inhibitor of the enzyme activity [67].

Additionally, antibody-based assays used in the described investigations differ in the binding site and epitope recognition. It is plausible to infer that distinct forms of BACE1 may have been explored in previous studies.

Moreover, recent research suggests that the existence of multiple enzyme isoforms could affect the correct estimation of BACE1 concentration. It is worth noting that some spliced forms do not have APP-cleaving activity and that it is not known which (and whether) specific forms vary in AD [55]. The same study shows that other enzymes, detectable in CSF, such as cathepsin B, meprin β, and BACE2, could exert β-secretase activity.

Some studies employed assays based on polyclonal antibodies [34, 68], which do not support the reproducibility of the assays on the longer term (cfr. long-term supply of antibodies with steady characteristics, lot-to-lot consistency of the assay, etc.). ELISA-based platforms, however, may have non-specificity due to weak polyclonal antibodies, and this can cause a discrepancy in data variation between different groups. The stability of BACE1 protein concentrations measured by sandwich ELISA shows limited change under standard storage and freeze/thaw conditions [69].

It will be crucial to develop and standardize the most appropriate methodologies, to understand the corresponding readout, and eventually focusing on CoU qualification to establish the potential role of BACE1 as an AD biomarker. While the activity-based measurements encounter low specificity, analyzing protein concentrations should consider the various known BACE1 protein isoforms also generated by post-translational modifications, membrane association, and truncated fragments [65]. Indeed, post-translational modifications influence the rate of activity of BACE1, thus accounting for a non-linear association with BACE1 gene expression levels [3].

A relatively easy step forward to increase specificity and analytical robustness of BACE1 biomarker assessment is to use immunoassays with well-defined antibodies. In this regard, an immunoassay based on two monoclonal antibodies (mAbs) has been established. It consists of a clone 10B8 that recognizes BACE1 within its extracellular, active domain (aa46-240) (aa numbering of human BACE1) and, as a complementary monoclonal antibody, clone 5G7, recognizing cleaved, non-membrane-bound BACE1 via a conformational epitope (aa299-312, a helical region of BACE1, and a free C-terminal Q386 end). In the first explorative study with this immunoassay, significant differences were observed in the CSF from AD patients compared to control individuals [26].

Although still at an initial stage, innovative molecular imaging for BACE1 assessment is under development. In line with the high-affinity of BACE1 inhibitors, recent efforts to develop brain-penetrant PET radiotracers, such as the highly potent selective aminothiazine inhibitor, PF-06684511 have been made [70].

As of other AD PET biomarkers, PET BACE1 ligands can investigate regional patterns of BACE1 activity and monitor BACE1 inhibitor regional brain effects.

In summary, pre-analytical and analytical protocols for BACE1, as well as other biomarkers for AD, should be harmonized and then standardized at a global scale to drastically reduce inter-study and longitudinal variability and eventually speed up the validation and qualification process of BACE1 biomarker candidates. The validation process will be facilitated not only by internationally accepted general requirements for the competence of testing and calibration laboratories but also by recently proposed standard operating procedures (SOPs) for Alzheimer’s biomarkers, including BACE1 (see Table 3 for specific information).

**Table 3:** Proposed stepwise validation path for Alzheimer’s disease biomarkers

Parameter	Definition
**Robustness**	The ability of a method to remain unaffected by small variations in method parameters
**Precision**	The closeness of agreement between independent test results obtained under stipulated conditions
**Trueness**	The closeness of agreement between the average value obtained from an extensive series of test results and an accepted reference value
**Uncertainty**	A parameter associated with the result of a measurement that characterizes the dispersion of the values could reasonably be attributed to the measurand
**Limits of quantification**	Highest and lowest concentrations of analyte that have been demonstrated to be measurable with acceptable levels of precision and accuracy
**Dilutional linearity**	Dilutional linearity is performed to demonstrate that a sample with a spiked concentration above the ULOQ can be diluted to a concentration within the working range and still give a reliable result
**Parallelism**	Relative accuracy from recovery tests on the biological matrix or diluted matrix against the calibrators in a substitute matrix
**Recovery**	The recovery of an analyte in an assay is the detector response obtained from an amount of the analyte added to and extracted from the biological matrix, compared to the detector response obtained for the true concentration of the analyte in the solvent
**Selectivity**	The ability of the bioanalytical method to measure and differentiate the analytes in the presence of components that may be expected to be present
**Sample stability**	The chemical stability of an analyte in a given matrix under specific conditions for given time intervals

Note: A committee, within the international research framework BIOMARKAPD, recently convened to propose the ten key requirements to fulfill within a step-by-step validation process. The BIOMARKAPD project aims for the standardization of biomarker measurements for AD and Parkinson’s disease (PD), including pre-analytical and analytical procedures, assay validation, and development of reference measurement procedures (RMP) and certified reference materials (CRM) for harmonization of results across assay formats and laboratories. The table captures stepwise standard operating procedures (SOP)

Table adapted from [71]

## Conclusions: challenges and perspectives

A potential explanation for the observed cognitive worsening in some of the previously reported late-stage BACE1 inhibitor trials may be related to insufficient APP substrate specificity. Other physiologically relevant BACE1 substrates may have been inhibited; some of these are involved in neuroplasticity, repair, and synaptic pathways. It may also be possible that BACE1-mediated APP processing could have been inhibited too strongly impairing physiological APP turnover or alternative APP-processing pathways may have been induced. More extensive research is needed to answer these questions.

Non-clinical, translational studies have shown that BACE1 activity is a relevant facilitator of axonal sprouting, dendritic remodeling, and synaptic plasticity, through both amyloidogenic and non-amyloidogenic pathways [3, 7]. In this regard, complete suppression of BACE1 enzymatic activity may substantially impair adult hippocampal neurogenesis [72, 73], which is a crucial mechanism for hippocampal synaptic plasticity and essential for memory formation and learning [72, 73].

Experimental studies in adult conditional BACE1 knockout mice indicated that pharmacological BACE1 inhibition might disrupt the organization of axonal pathways in the hippocampus [3]. Regarding peripheral toxicity, most of the BACE1 inhibitors block the activity of BACE2 as well, a close homolog of BACE1, which may cause additional unwanted on-target side effects.

Among several open scientific issues, we highlight the question of whether the negative correlation between CSF BACE1 enzymatic activity and degree of hippocampal atrophy may be primarily induced by the BACE1 downstream amyloidogenic effects or on affected synaptic pathways as well. The interrelation between BACE1 and progressive neurodegeneration deserves further investigation. Neurodegeneration biomarker panels, including tau and NFL proteins, provide partially differential information of related pathophysiological processes [74].

There is evidence of a complex interaction between the amyloidogenic pathway and other pathomechanistic alterations of AD, including neuroinflammation. TNF receptor (TNFR1) knockout mice show decreased Aβ peptides and cerebral accumulation of amyloid plaques through regulation of BACE1 gene expression via the nuclear factor κB (NF-κB) pathway [75, 76].

Addressing these scientific questions will provide key pathophysiological insights and facilitate the implementation of standardized drug-biomarker co-development programs that are necessary to achieve successful BACE1 targeting strategies for precision medicine.

BACE1, either in CSF or blood and either activity or protein concentration, does not show a remarkable performance as a clinical diagnostic or pathognomonic AD biomarker. However, whether BACE1 biomarkers could increase diagnostic accuracy if combined with AD core biomarkers has been poorly investigated. Given the preliminary evidence about the association between BACE1 and Aβ biomarkers as well as neurodegeneration biomarkers (namely hippocampal volumes and t-tau) and synaptic biomarkers (neurogranin), we support the investigation of BACE1 parameters in combination with the AD core biomarker panel, across different matrixes such as CFS and blood, to assess diagnostic performance in the AD continuum (preclinical, prodromal, dementia stages). Association studies indicate that BACE1 biomarkers may be useful for COU in a clinical trial setting, including proof of mechanism, treatment response, and safety assessment in clinical trials, as well as COU in a clinical practice setting such as prognostic evaluation in MCI individuals. From a therapeutic perspective, BACE1 inhibitors dosing could be personalized to engage targets based on direct concentrations and activity rate measurements from individual bodily fluids [27, 74]. Furthermore, the reduction of cleavage products, such as sAPPβ, or enriched alternatively processed peptides such as Aβ5-X, which are correlated to BACE1 inhibition, could be used to monitor target engagement and optimize efficacy [15, 77]. Future investigations using combinatorial strategies and biomarker-guided or personalized dose selection may allow the application of lower doses with an optimized specificity for BACE1 over BACE2 [3].

Developing assays for the analysis of Aβ species, including bioactive oligomers, provide a more profound understanding of human pathophysiology and the relationship between BACE1 and elements of the downstream amyloid pathway, from Aβ species supporting synaptic and homeostatic functions to more bioactive and toxic species [3, 78].

While experimental studies foster knowledge generation of BACE1’s complex biology, including synaptic homeostasis, BACE1 fluid biomarker development still needs to transition through the validation and standardization process with harmonized pre-analytical and analytical protocols.

From a pharmacological standpoint, BACE1 biomarkers are expected to be essential components of drug-biomarker co-development programs supporting successful outcome generations and lowering drug attrition rates in pipelines targeting BACE1. From a medical practice standpoint, liquid biopsy with first availability of CSF followed by maturation of blood-based BACE1 biomarkers [79] may expand the current descriptive A/T/N classification system into developing a comprehensive and integrative biological staging model of AD.

## Data Availability

Not applicable
